# Septic arthritis of the pubic symphysis: a case report

**DOI:** 10.11604/pamj.2014.18.149.4421

**Published:** 2014-06-17

**Authors:** Imane El Mezouar, Fatema Zahra Abourazzak, Samia Mansouri, Taoufik Harzy

**Affiliations:** 1 Rheumatology Department, Medical School, Sidi Mohammed Ibn Abdellah University, Hassan II University Hospital, Fez, Morocco

**Keywords:** Septic arthritis, pubic symphysis, osteomyelitis

## Abstract

Septic arthritis of the pubic symphysis, so called osteomyelitis pubis is the infection which involves pubic symphysis and its joint. It is a rare condition, representing less than one percent of all cases of osteomyelitis. It affects most frequently young athletes and women undergoing gynecologic or urologic surgery. It presents itself with fever and pubic pain which irradiates to the genitals and increases when hip is mobilized, and this fact produces gait claudication. Diagnosis is based on clinic supported by microbiologic culture results, image methods, and proteins augment during acute phase. The etiologic agents most commonly found are Staphylococcus aureus, followed by gram-negative bacilli, and polymicrobial infection in recent pelvis surgery. The antibiotic treatment is adjusted depending on the microbiological diagnosis, adding NSAIDs, and bed rest. We report a 16 year-old male presenting with pubic pain and fever. Magnetic resonance imagery showed arthritis of the pubic symphysis. The patient was treated with antibiotics with a good clinical response.

## Introduction

Septic arthritis of the pubic symphysis is rare. The symptoms can mimic a myriad of pathologies masking the true diagnosis. Radiographic signs can be delayed or undetected in certain modalities of radiological investigation. Therefore, the diagnosis can be missed and treatment delayed. We describe one such case presenting to our institution which highlights these difficulties and reiterates the importance of maintaining a high level of suspicion based on the clinical symptoms and key points in the history to ensure prompt treatment and avoid severe complications.

## Patient and observation

**Figure 1:**
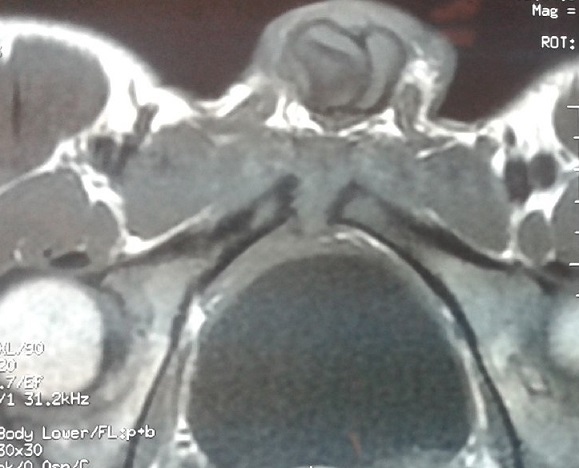
The magnetic resonance imagery (MRI) T1 of the pelvis showed bone oedema of symphysis pubis and abdominal muscles

**Figure 2:**
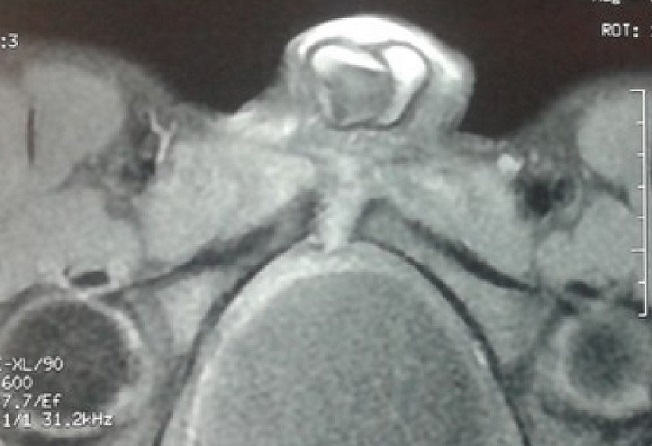
The magnetic resonance imagery (MRI) T1 of the pelvis showed bone oedema of symphysis pubis and abdominal muscles after gadolinium injection

A 16-year-old man presented with a one month history of fever, pubic pain which irradiates to the genitals and increases when hip is mobilized, and this fact produces gait claudicating. The symptoms started a few days after playing a football match, though the patient denied any history of trauma. He had a temperature of 38.5°C and a normal testicular and abdominal examination. The C reactive protein had risen to 12 mg/l with an erythrocyte sedimentation rate of 25 mm/h and a white blood cell of 16410 per mm3. Echocardiogram was normal and autoimmune screen was negative. The blood culture was negative. The magnetic resonance imagery (MRI) of the pelvis showed a bone oedema of symphysis pubis and abdominal muscles (Figure 1, Figure 2). The puncture guided by computed tomography (CT) of the articulation of pubis returned of Staphylococcus aureus methicillin-susceptible (SAMS). The patient was prescribed intravenous flucloxacillin 1 g six times a day for six weeks and discharged with further four months of oral flucloxacillin at 1g three times a day on microbiologist's advice. A repeat MRI after four months showed a complete resolution.

## Discussion

The pubic symphysis is the insertion point of the hip adductors and rectus abdominus muscles. Inflammation and infection at this site is a recognized complication in athletes whose activities consist of repetitive over-adduction and twisting such as that experienced by footballers [1, 2]. It presents with pain on hip abduction, pubic tenderness, pain while walking, difficulty weight-bearing and testicular, suprapubic and abdominal pain [1]. In the context of no overt sepsis this is described as osteitis pubis and is in itself a rare diagnosis [3]. An even rarer diagnosis is septic arthritis which can often be misdiagnosed as osteitis pubis but is importantly associated with sudden onset of pain, fever and positive blood cultures or aspirate [1, 4]. There is some debate as to whether these two pathologies represent a spectrum of disease with septic arthritis occurring in those predisposed to repetitive strain and an existing osteitis pubis [1, 2]. S aureus is commonly implicated in septic arthritis occurring in athletes [2, 4]. The pathogenesis is thought to be due to microtrauma with repetitive movement during sports that makes it susceptible to seeding of S *aureus* (which is transiently present in the body) and subsequent colonization [5]. MRI scanning is the most reliable method of detecting the disease as the changes on CT scan and radiograph can be delayed [2]. In our case, the presenting symptoms and signs were typical of a pubic symphysis septic arthritis and highlights that this disease can be associated with varying levels of sporting activity. The absence of early radiological and CT signs suggests that the septic arthritis had arisen spontaneously. Retropubic abscess formation is secondary to the septic arthritis of the pubic symphysis [6, 7]. The ideal treatment is a prolonged course of intravenous and oral antibiotics and abscess drainage if possible [1, 3]. Awareness and early recognition can prevent disease progression and unnecessary invasive treatment [8, 9].

## Conclusion

Septic arthritis of the pubic symphysis is a rare condition. Diagnosis is based on clinic supported by microbiologic culture results, image methods, and proteins augment during acute phase. The antibiotic treatment is adjusted depending on the microbiological diagnosis, adding NSAIDs, and bed rest.
